# Metagenomic analysis of bile acid biotransformation by gut microbiota in wild birds

**DOI:** 10.1016/j.psj.2025.105956

**Published:** 2025-10-10

**Authors:** Meng-Ting Yang, Lin-Hong Xie, Ling Wang, Ying-Qian Gao, Rui Liu, He Ma, Cong-Cong Lei, Jing Jiang, Jin-Wen Su, Xiao-Xuan Zhang, Hong-Bo Ni, Fu-Long Nan

**Affiliations:** a College of Veterinary Medicine, Qingdao Agricultural University, Qingdao, Shandong Province, PR China; b College of Life Sciences, Changchun Sci-Tech University, Shuangyang, Jilin Province, PR China; c Center of Prevention and Control Biological Disaster, State Forestry and Grassland Administration, Shenyang, Liaoning Province, PR China; d School Hospital, Qingdao Agricultural University, Qingdao, Shandong Province, PR China

**Keywords:** Bile acid metabolism, Environmental factor, Gut microbiota, Migratory and resident bird, Wild bird

## Abstract

Although gut microbiota-mediated bile acid (BA) metabolism is well characterized in mammals, its mechanisms in wild birds remain largely unknown, hindering our understanding of their ecological adaptation and health. In this study, metagenomic analysis was performed on 10,455 metagenome-assembled genomes (MAGs) derived from 718 wild bird gut samples, from which 1,034 high-quality non-redundant MAGs were selected for further analysis. Functional annotation analysis identified 755 MAGs encoding genes associated with BA biotransformation pathways, primarily derived from the phyla Bacillota_A, Bacteroidota, and Bacillota, with dominant genera including *Helicobacter_G* and *Ligilactobacillus*. Subsequent genomic analysis identified 379 MAGs encoding bile salt hydrolase (BSH), with phylogenetic classification demonstrating predominant affiliation to the Bacteroidota and Bacillota_A phyla. Compared to the BSH-producing microbiota in the human and chicken gut, the phylum Bacillota exhibited a notably higher relative abundance in wild birds. Within the wild bird gut microbiome, *Helicobacter_G* was identified as the predominant BSH-encoding genus, whereas its relative abundance was substantially lower in both humans and chickens. Moreover, migratory birds (MB) displayed significantly higher diversity of BA biotransformation genes than resident birds (RB), with *Helicobacter_G* being notably enriched at the genus level in MB, potentially associated with their heightened energy and nutritional demands during migration. Notably, in addition to residency status, host species emerged as the most influential factor shaping the compositional variation of BA biotransformation genes, followed by environmental factors and dietary habits. In summary, this study systematically elucidates the potential functions of gut microbiota in BA metabolism and their close associations with host ecological traits in wild birds, not only advancing our understanding of host-microbe interactions and metabolic adaptation mechanisms but also providing a theoretical foundation for future interventions targeting gut microbiota to improve wildlife health.

## Introduction

The gut microbiota constitutes a critical symbiotic system that regulates host metabolism, maintains immune homeostasis, and supports overall health (Li et al., 2022; Rooks and Garrett, 2016; Shang, et al., 2025; Shreiner, et al., 2015). Bile acids (BAs), as the end products of cholesterol metabolism, not only play a critical role in the digestion and absorption of dietary lipids, but also act as signaling molecules that regulate various host physiological processes through their interactions with the gut microbiota (Babu, et al., 2022; Festa et al., 2017; Wang, et al., 2021). Through interactions with the gut microbiota, BAs modulate host energy metabolism, immune responses, and the integrity of the intestinal barrier (Yang, et al., 2021). Gut microbes convert primary BAs into secondary BAs with diverse biological activities through various enzymatic reactions, including deconjugation, dehydrogenation, and dihydroxylation, thereby modulating multiple physiological functions of the host (Guzior and Quinn, 2021; Sharma, et al., 2022). Dysregulation of BA metabolism can lead to metabolic syndrome, inflammatory bowel disease, and other disorders(de Bruijn, et al., 2020; Duboc, et al., 2013). Therefore, BA metabolism has become a pivotal focus in elucidating the mechanisms underlying host–microbiota interactions.

Although BA metabolism and its interaction with the gut microbiota have been extensively studied in mammals, relevant research in birds—particularly in wild species—remains limited (Jia, et al., 2018; Wang, et al., 2019). Wild birds, due to their unique dietary habits, migratory behaviors, and diverse habitats, may have evolved BA metabolic and regulatory mechanisms distinct from those of mammals (Mi, et al., 2023; Sun, et al., 2022). However, systematic understanding of the functional roles of gut microbiota in avian BA metabolism and their relationship with host ecological adaptation is still lacking, which greatly limits our insight into avian physiological adaptation strategies. A key mechanism underlying this host-microbiota interaction is microbial BA biotransformation, the enzymatic conversion of host-derived primary BAs into secondary BAs with distinct signaling and biological properties.

In this study, we aimed to systematically explore the BA biotransformation potential of gut microbiota in wild birds and investigate the ecological and host-related factors shaping this capacity. To achieve this, we performed large-scale metagenomic analysis based on 10,455 metagenome-assembled genomes (MAGs) reconstructed from 718 gut samples collected from diverse wild bird species. We then focused on identifying microbial taxa and genes associated with BA biotransformation, including bile salt hydrolase (BSH), and examined their phylogenetic distribution, functional diversity, and host-specific patterns. By comparing the BA metabolism-related microbiota between migratory birds (MB) and resident birds (RB), we further assessed the potential ecological implications of these microbial functions. Furthermore, we examined how host species and environmental factors influence both the composition of BA biotransformation genes and the taxonomic profiles of the contributing microbial taxa. This comprehensive analysis provides new insights into the gut microbial contributions to metabolic adaptation and ecological fitness in wild birds.

## Materials and methods

### Data collection and preprocessing of genomes

In this study, we utilized 10,455 MAGs derived from the gut microbiota of wild birds, which were sourced from the Figshare database (https://doi.org/10.6084/m9.figshare.28504238). These MAGs correspond to 718 publicly available gut metagenomic datasets from wild birds, encompassing a broad taxonomic range that includes 14 avian orders, 25 families, 53 genera, and 92 species (Supplementary Table 1).

Quality control of raw sequencing data from 718 wild bird gut metagenomes was performed using fastp (v0.23.0) (Chen, et al., 2018) with the following parameters: -q 20 -u 30 -n 5 -y -y 30 -l 80 -trim_poly_g. To minimize host gene interference, Bowtie2 (v2.5.0) (Langmead and Salzberg, 2012) was employed to remove host genomic sequences from the quality-controlled reads (parameters: –end-to-end –mm –fast). Clean reads were then obtained for subsequent analysis.

### Genomes quality evaluation, taxonomic classification, and gene prediction

The completeness and contamination levels of the 10,455 collected genomes were evaluated using ChechM2 (v1.0.1) (Chklovski, et al., 2023). Genomes that met the selection criteria of ≥ 80 % completeness and ≤ 5 % contamination were advanced to subsequent analyses. Following this initial screening, 1,381 genomes were retained. These genomes were then subjected to taxonomic classification using the classify_wf workflow in GTDB-Tk (v2.3.2) (Chaumeil, et al., 2022). Subsequently, genome clustering was performed with dRep (v3.4.5) (Olm, et al., 2017), utilizing the parameters -pa 0.9, -sa 0.99, -nc 0.30, -cm larger, and –S_algorithm fastANI. Based on these criteria, a total of 1,034 genomes were selected for further analysis. Finally, open reading frames (ORFs) were predicted for these genomes using Prodigal (v2.6.3) (Hyatt, et al., 2010) with the parameter -p single. A maximum likelihood tree was constructed using PhyloPhlAn (v3.0.67) (Asnicar, et al., 2020).

### Functional annotation

BLASTP (v2.13.0) (Altschul, et al., 1990) was used to perform functional annotation of 1,034 MAGs against the Kyoto Encyclopedia of Genes and Genomes (KEGG) database. High-quality reads (20 million reads) from 718 wild bird gut metagenomes were mapped to a non-redundant gene catalog using Bowtie2 (v2.5.0) (Langmead and Salzberg, 2012). Subsequently, the read counts for each sample were converted to transcripts per million (TPM). Annotated results were further selected, and 15 KOs involved in BAs metabolism, include K00076, K01442, K07007, K15868, K15869, K15870, K15871, K15872, K15873, K15874, K22604, K22605, K22606, K22607, and K23231. Based on the information available for the genes encoding these KOs, their origin in genomes was determined and their copy numbers in the genome were calculated.

### Correlation analysis between microbial species and bile acid transformation genes

To investigate the relationships between specific microbial taxa and the genetic potential for BA transformation, we performed a Spearman rank correlation analysis. The analysis was conducted between the abundance profiles of bacterial species (derived from the 1,034 MAGs) and the abundance profiles of key BA-transforming genes (KEGG orthologs). Only correlations with an absolute Spearman's |ρ| > 0.5 and a false discovery rate (FDR) adjusted p-value (Benjamini-Hochberg method) < 0.05 were considered statistically significant.

### Environmental data collection

To obtain environmental background information for each sample point, we extracted four high-resolution climate factors from the CHELSA database (v2.1) (Karger, et al., 2023): mean annual temperature (BIO1), temperature seasonality (BIO4), annual precipitation (BIO12), and precipitation seasonality (BIO15). We processed and extracted raster data using the terra package (v1.7-65) in R, converting BIO1 values to Celsius (°C). Additionally, we acquired elevation data based on AWS Terrain Tiles using the elevatr package (v0.99.0) and standardized the geographical coordinates of all sampling points with the help of the sf package (v1.0-14).

### Statistical analyses and visualization

All statistical analyses were conducted in R (v4.2.2). The visualization of the phylogenetic tree was conducted on the iTOL (v6.9.1) platform (Letunic and Bork, 2021). Taxonomic and functional gene abundance data were used to calculate the Richness and Shannon indices. β-diversity was assessed through Principal Coordinate Analysis (PCoA) based on Bray-Curtis distance, and group differences were evaluated using permutational multivariate analysis of variance (PERMANOVA). The Wilcoxon rank-sum test was utilized to evaluate the significance of differences in diversity indices, taxonomic units, and functional gene feature abundances among groups. The R package "vegan" (v2.6-4) was used to generate rarefaction curves and conduct Redundancy Analysis (RDA). Sankey plot was visualized using the ‘ggsankey’ package (v0.0.9). The significant correlations were visualized as a heatmap using the pheatmap package (v1.0.13) in R. All other visualizations were generated using the ‘ggplot2’ package (v4.2.3).

## Result

### Collection of wild bird gut microbiome MAGs data

**Fig. 1:**
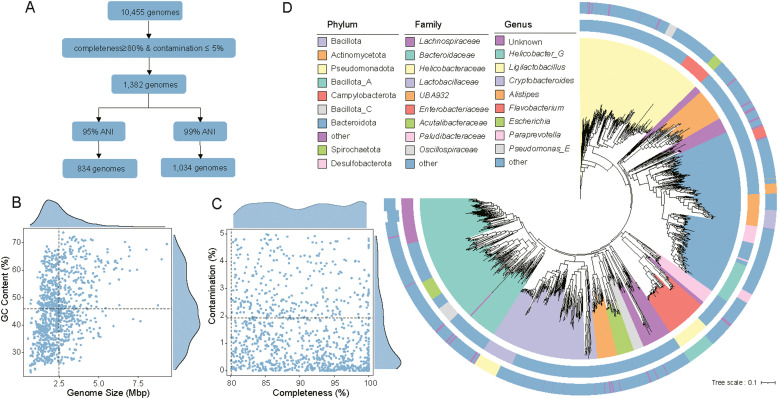
**Genomic information collected for analysis. A:** Presents a flowchart illustrating genome screening and processing in this study, including quality assessment and removal of redundant data steps. **B-C:** Display basic statistical characteristics of 1,034 genomes, encompassing genome size, GC content, integrity, and contamination level. **D:** Depicts a phylogenetic tree of 1,034 genomes constructed based on the maximum likelihood method, revealing their taxonomic distribution at the phylum and family levels.

A total of 1,034 non-redundant MAGs were obtained for further analysis (Fig. 1A). The genomic sizes range from 0.52 to 9.32 megabases (Mbp) per genome (with an average of 2.49 Mbp per genome), and the GC content ranges from 22.84 % to 73.72 % (with an average of 45.91 %) (Fig. 1B). The mean completeness of these genomes was 90.61 %, with an average contamination of 1.35 % (Fig. 1C).

The taxonomic analysis conducted using the GTDB-Tk database revealed that these genomes are categorized into 29 phyla, 41 classes, 97 orders, 194 families, 424 genera, and 262 species(Fig. 1D). Among them, 695 genomes, accounting for 67 % of the total, could not be precisely assigned to known species. In terms of abundance, the most prevalent phyla are Bacteroidota (227 genomes, 22.0 %), Bacillota_A (218 genomes, 21.1 %), Pseudomonadota (177 genomes, 17.1 %), and Bacillota (148 genomes, 14.3 %). At the family level, the most abundant families include Lachnospiraceae (56 genomes, 5.4 %), Bacteroidaceae (50 genomes, 4.8 %), Helicobacteraceae (41 genomes, 4.0 %), Faecalibacillaceae (39 genomes, 3.8 %), and UBA932 (39 genomes, 3.8 %).

### Analysis of the involvement of MAGs in bile acid metabolism pathways and key enzymes

**Fig. 2:**
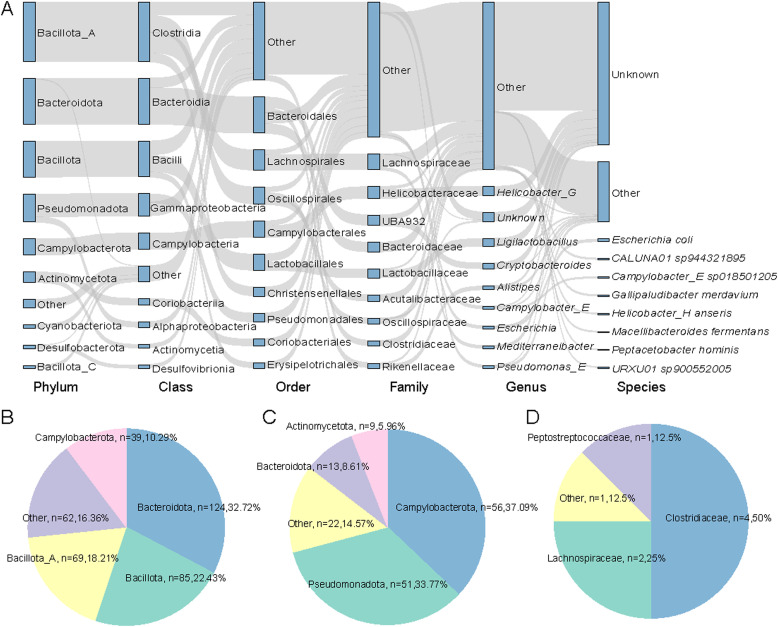
**Bile Acid (BA) metabolism Pathways in 755 Genomes. A:**Illustrates the microbial community composition and relative abundance across different taxonomic levels—phylum, class, order, family, genus, and species—within the associated genomes. **B-D:** Respectively depict the prevalence of genes encoding BSH (B), 7α-HSDH (C), and baiCD (D) within the dataset.

To systematically elucidate the BA biosynthetic network mediated by the gut microbiota of wild avian species, this study conducted functional annotation of MAGs using the KEGG database. Through comprehensive annotation analysis, 755 MAGs (representing over 73 % of the total) were identified as participating in BA transformation pathways. Among these, 1,337 genes encoding BA-metabolizing enzymes were characterized, encompassing critical enzymatic steps such as BA side-chain decoupling, hydroxyl oxidation, and steroid nucleus dihydroxylation (Supplementary Table 2). Bacteria from the Bacillota_A phylum (mainly Clostridia) were the dominant carriers BAs KO, followed by Bacteroidota and Bacillota (Fig. 2A).

In these MAGs, we identified 379 genomes encoding BSH (choloylglycine hydrolase [K01442; EC:3.5.1.24]), an enzyme responsible for hydrolyzing conjugated bile salts into deconjugated BAs. These BSH-encoding genomes were taxonomically assigned to 10 phyla, with the majority affiliated with Bacteroidota (*n* = 124), followed by Bacillota (*n* = 85) and Bacillota_A (*n* = 69) (Fig. 2B). This indicates that a wide range of microbial taxa in the gut microbiota of wild birds possess the capacity to hydrolyze bile salts. At the family level, the BSH-carrying genomes were predominantly classified into Bacteroidaceae (*n* = 34), Helicobacteraceae (*n* = 34), and UBA932 (*n* = 31). Genus-level assignments showed that most genomes belonged to *Helicobacter_G* (*n* = 29) and *Ligilactobacillus* (*n* = 26) (Supplementary Table 2). In addition, our analysis revealed that only 10 phyla encoded 7α-HSDH (7-alpha-hydroxysteroid dehydrogenase [K00076; EC:1.1.1.159]), an NAD(P)+-dependent enzyme that oxidizes the hydroxyl groups of deconjugated BAs. These 7α-HSDH-encoding genomes were mainly derived from Campylobacterota (*n* = 56) and Pseudomonadota (*n* = 51) (Fig. 2C).

Moreover, only a single phylum, Bacillota_A (*n* = 8) (Fig. 2D), was found to encode baiCD (3-oxocholoyl-CoA 4-desaturase; [K15870; EC:1.3.1.115]), a key enzyme involved in the 7α-dehydroxylation pathway of BA transformation. These genomes were assigned to the families Clostridiaceae (*n* = 4), Lachnospiraceae (*n* = 2), Cellulosilyticaceae (*n* = 1), and Peptostreptococcaceae (*n* = 1).

Correlation analysis between microbial species and bile acid metabolism enzyme genes revealed a distinct specificity in the distribution of genes encoding the key enzymes 7α-HSDH and BSH. The vast majority of species showed a significant correlation with only one of the two enzymes, suggesting potential functional specialization. A notable exception was *CALURLO1 sp044326915*, which exhibited strong positive correlations with both 7α-HSDH (correlation coefficient: 0.609) and BSH (correlation coefficient: 0.519). This implies that this species may possess the metabolic capability to simultaneously deconjugate primary bile acids and subsequently oxidize the 7α‑hydroxy group.

In contrast, *Cereibacter changlensis* displayed a specific high correlation with 7α-HSDH (0.553) but not with BSH. Conversely, several other species (e.g., *Bacteroides stercoris* and *Bifidobacterium pullorum_B*) were correlated solely with BSH or other metabolic pathways, showing no association with 7α-HSDH. This distribution pattern indicates that deconjugation and 7α‑hydroxy oxidation redox reactions of bile acids are generally governed by distinct microbial functional groups within the gut microbiota (Supplementary Fig. 1A).

### BA metabolism of gut microbiota specific to wild birds

**Fig. 3:**
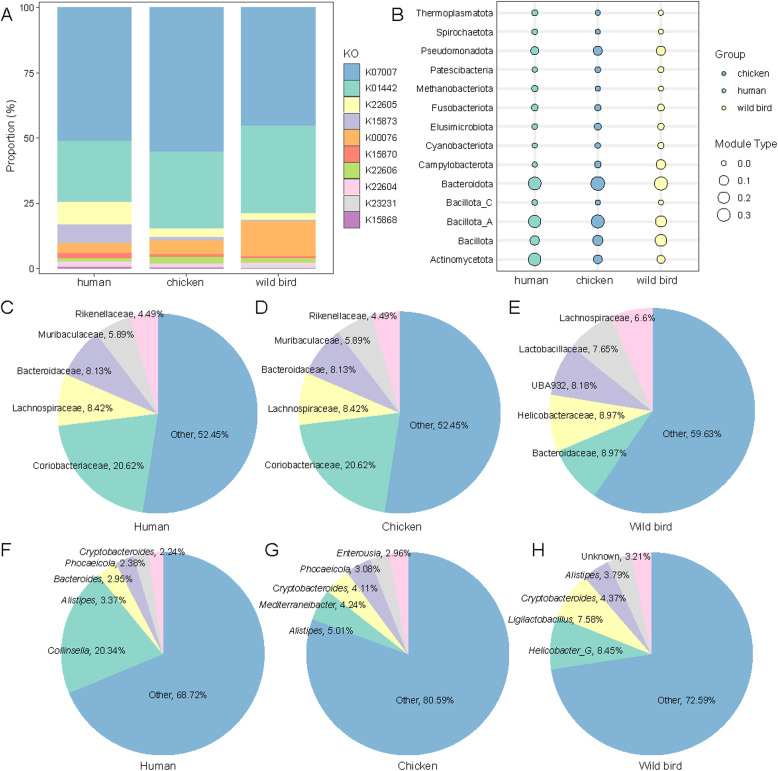
**Bile Acid (BA) metabolism of the gut microbiota in humans, chickens, and wild birds is analyzed. A:**Comparative analysis of genes related to BA metabolism in the gut microbiota of humans, chickens, and wild birds. **B-H:** Taxonomic classification of MAGs harboring the bsh gene in the human, chicken, and wild bird gut microbiota at the phylum(B), family(C-E), and genus levels(F-H).

To assess the functional specificity of BA metabolism in the gut microbiota of wild birds, we performed a systematic comparative analysis between 755 gut microbial genomes recovered in this study and previously reported MAGs from humans (2,294 MAGs) (Nayfach, et al., 2019) and chickens (2,113 MAGs) (Supplementary Table 3). Taxonomic classification and functional annotation revealed a total of 3,499 BA-related KEGG orthologs (KOs) in the human gut microbiome, while 2,897 BA-related KOs were identified in the chicken gut microbiome. Notably, the relative abundance of 7α-HSDH (K00076) was higher in the gut MAGs of wild birds compared to those of the other two groups. Apart from this notable difference, the relative abundances of other major BA-transforming KOs were largely comparable between wild birds and domestic chickens (Fig. 3A).

Regarding BA deconjugation, BSH was broadly distributed in a host-independent manner, with 31.08 % of human intestinal MAGs, 36.82 % of chicken intestinal MAGs, and 36.65 % of wild bird intestinal MAGs encoding BSH. Bacteroidota and Bacillota_A were the dominant phyla across all three host groups, while Actinomycetota exhibited the highest relative abundance in the human gut. This elevated abundance of Actinomycetota clearly distinguishes the human gut microbiome from that of both wild birds and domestic chickens (Fig. 3B). At the family level, BSH-carrying MAGs were predominantly assigned to Coriobacteriaceae in humans (20.62 %), Lachnospiraceae in chickens (13.37 %), and Bacteroidaceae (8.97 %) and Helicobacteraceae (8.97 %) in wild birds (Fig. 3C-3E). *Helicobacter_G* (8.45 %) was the most prevalent BSH-encoding genus in the wild bird gut, whereas its prevalence was much lower in humans and chickens (Fig. 3F-3H).

Taken together, although BSH genes are widely distributed in the intestinal microbiota of humans, chickens, and wild birds, their taxonomic affiliations display distinct host-specific patterns. In wild birds, *Helicobacter_G* and *Ligilactobacillus* were the dominant BSH-carrying genera, whereas in humans and chickens, *Collinsella* and *Alistipes* were more prevalent, respectively. These findings suggest that the potential for BA deconjugation is driven by host-specific microbial lineages, with wild birds, humans, and chickens each harboring distinct, dominant taxa responsible for this function.

### Comparative analysis of BA biotransformation genes in gut microbiota of MB and RB

**Fig. 4:**
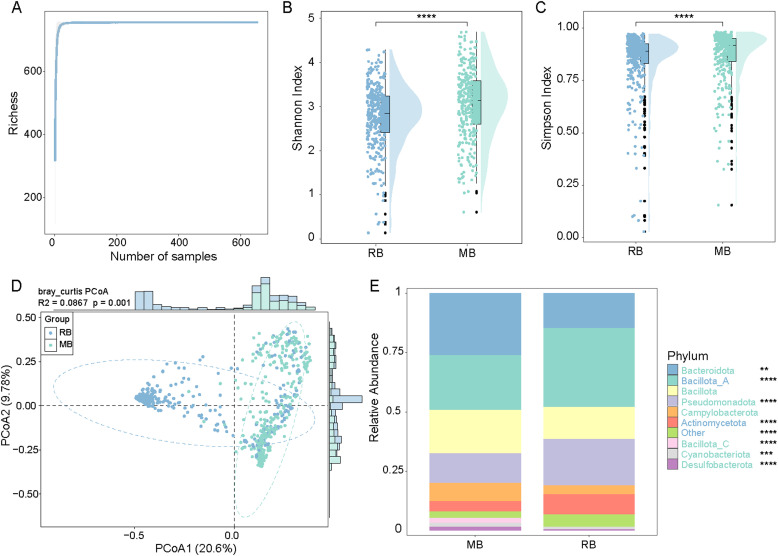
**Comparative analysis of BA (BA) biotransformation gene diversity and gut microbial composition between MB and RB groups. A:**Rarefaction curve of BA biotransformation genes in the gut microbiota. **B-C:** α-diversity comparisons between MB and RB based on Shannon index (B) and Simpson index (C). **D:** Principal coordinate analysis (PCoA) based on Bray–Curtis distances. **E:** Phylum-level relative abundance of microbial communities in MB and RB groups. Differentially enriched phyla are annotated with significance levels: *P* < 0.01 (**), *P* < 0.001 (***), *P* < 0.0001 (****).

The divergence in survival tactics and energy expenditure between migratory birds (MB) and resident birds (RB) may exert a profound impact on the gut microbiota, potentially modulating the capacity for BA biotransformation within these microbial communities. It is well-established that primary bile acids are synthesized by the host liver, while the gut microbiota is responsible for their biotransformation into secondary bile acids (Guzior and Quinn, 2021). To elucidate this phenomenon, the present study conducted an exhaustive comparative analysis of the genetic variations associated with BA biotransformation in the gut microbiota of MB and RB. Utilizing rarefaction curve analysis, it was observed that the diversity of BA biosynthetic genes within the gut microbial communities asymptotically approached saturation as the sample size increased (Fig. 4A).

To thoroughly evaluate microbial diversity between the two sample groups, we calculated α-diversity indices (Shannon and Simpson indices) and performed principal coordinate analysis (PCoA) based on Bray-Curtis distance. The results demonstrated that the MB group exhibited significantly higher Shannon and Simpson indices compared to the RB group (Fig. 4B and 4C; *P* < 0.0001), indicating greater species richness and community evenness in the MB group. PCoA revealed clear separation between the two groups in community structure (PCo1: 20.6 %, PCo2: 9.78 %, cumulative explained variation: 30.38 %). Adonis analysis further confirmed the significant influence of grouping factors on community composition (Fig. 4D; R² = 0.0867, *p* = 0.001). In conclusion, the MB group showed superior α- and β-diversity compared to the RB group, suggesting distinct BA metabolic functions between these avian species.

We further characterized the gut microbiota associated with distinct BA biotransformation genotypes in wild birds. At the phylum level, the RB group was significantly enriched in Bacillota_A (*P* < 0.0001) and Actinomycetota(*P* < 0.0001), whereas the MB group was dominated by Bacteroidota(*P* < 0.01), Pseudomonadota(*P* < 0.0001), Bacillota_C(*P* < 0.0001), Cyanobacteriota(*P* < 0.001) and Desulfobacterota(*P* < 0.0001) (Fig. 4E). At the genus level, 116 genera were detected in the RB group, with Cryptobacteroides(*P* < 0.0001) and Eubacterium_R(*P* < 0.0001) were the significantly enriched; in contrast, the MB group comprised 194 genera, among which Ligilactobacillus(*P* < 0.0001), Mediterraneibacter(*P* < 0.0001), Clostridium(*P* < 0.0001), Escherichia(*P* < 0.0001) and Pseudomonas_E(*P* < 0.001) were the most enriched (Supplementary Fig. 1B). These compositional distinctions indicate that, beyond phylum-level community preferences, the RB and MB genotypes harbor genus-specific microbial assemblages with unique metabolic potentials, which may in turn modulate host BA profiles and related physiological processes.

### Influence of host species on BA biotransformation gene composition

Building on our earlier finding of pronounced differences between MB and RB in the diversity and origins of gut microbial BA biotransformation genes, we further assessed the potential regulatory roles of host species variation and environmental factors in driving these divergent characteristics. Then, we focused on six wild bird species, obtaining at least 19 samples per species (*n* = 374; Supplementary Table 4).

**Fig. 5:**
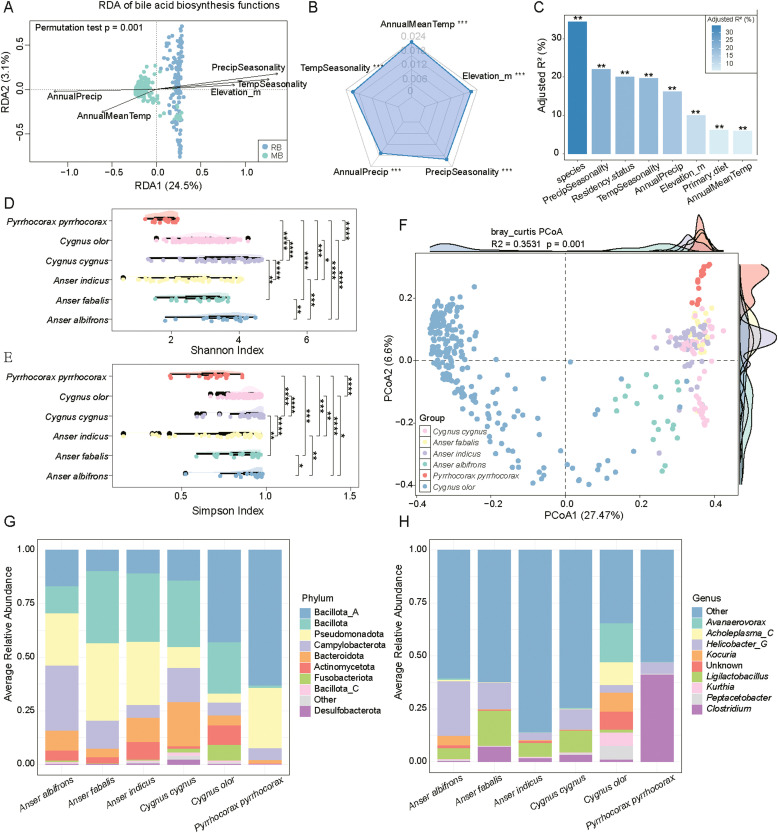
**Effects of host species and environmental factors on gut microbial bile acid biotransformation gene diversity and taxonomic composition in wild birds. A:**Redundancy analysis (RDA) of BA biotransformation functions based on environmental variables. **B:** Radar plot showing the marginal (independent) effects of five environmental variables on microbial functional composition, all of which are significant (*P* < 0.001). **C:** Bar plot of adjusted R² values from PERMANOVA showing that host species, precipitation seasonality, and residency status are the strongest contributors to functional variation (*P* < 0.01, *P* < 0.001). **d-E:** Differences in α-diversity (Shannon and Simpson indices) of BA biotransformation genes among six bird species. **F:** Principal coordinate analysis (PCoA) of BA biotransformation gene composition across species based on Bray–Curtis distances (R² = 0.3531, *P* = 0.001). **G-H:** Taxonomic composition of BA biotransformation genes at the phylum (G) and genus (H) levels.

Environmental factors significantly shape the structure of BA biotransformation functions in wild bird gut microbiota, as shown by redundancy analysis (RDA) and marginal effect tests (permutation test, *p* = 0.001). In the RDA biplot, the first constrained axis (RDA1, 24.5 %) distinctly separates RB and MB samples. Precipitation seasonality, temperature seasonality, and annual precipitation are the main drivers, with the former two aligned with the RB cluster and annual precipitation aligned with MB. Annual mean temperature and elevation also contribute, though to a lesser extent (Fig. 5A). The marginal effect radar plot quantifies each variable’s independent contribution. Annual mean temperature, elevation, precipitation seasonality, annual precipitation, and temperature seasonality are all significant after accounting for other variables (Fig. 5B; *P* < 0.001), indicating a joint role of these five factors in shaping functional microbial profiles across host groups. Using PERMANOVA for further quantification, we found that host species accounted for the largest proportion of variation in gene composition (adjusted R² = 34.43 %; Supplementary Table 5), followed by precipitation seasonality, residency status, and temperature seasonality. All these factors exhibited significant effects (*P* < 0.001; Fig. 5C).

In terms of functional diversity, significant differences in Shannon and Simpson indices were observed among the six host species (*P* < 0.01; Fig. 5D, 5E), with Cygnus cygnus exhibiting the highest diversity. PCoA based on Bray-Curtis distances further confirmed significant interspecies variation in gene composition (PERMANOVA, R² = 0.3531, *P* = 0.001; Fig. 5F). Taxonomic origin analysis of BA biotransformation genes revealed significant differences in the major contributing microbial phyla and genera across different host species. At the phylum level, Bacillota_A was identified as the predominant phylum, while the relative abundances of Pseudomonadota and Campylobacteria varied substantially among species (Fig. 5G). Notably, *Anser fabalis, Anser indicus*, and *Pyrrhocorax pyrrhocorax* exhibited higher proportions of Pseudomonadota. At the genus level, *Achromoplasma_C* and *Helicobacter_G* emerged as dominant genera. *Helicobacter_G* was more abundant in *Anser albifrons* and *Anser fabalis*, whereas *Achromoplasma_C* was predominant in *Cygnus olor* (Fig. 5H). Overall, the microbial community composition showed significant interspecies variation at both the phylum and genus levels, highlighting the substantial influence of host species on the functional composition and microbial origin of BA biotransformation genes.

## Discussion

As a unique group of non-model hosts with distinctive physiological structures and ecological behaviors, wild birds possess gut microbiota that may exhibit specialized functional differentiation in key physiological processes such as BA metabolism (Grond et al., 2018; Jiang, et al., 2020). However, the functional roles of their gut microbiota in BA metabolism and the potential ecological implications remain poorly understood. In this study, we performed quality filtering and dereplication on 10,445 MAGs, resulting in 1,034 non-redundant MAGs spanning 29 phyla and exhibiting broad phylogenetic diversity. Over two-thirds of these MAGs could not be classified into known species, indicating the presence of a vast number of uncharacterized microbial groups in the bird gut (Grond et al., 2018; Hird, 2017; Kohl, 2012). In contrast, the human and chicken gut microbiota showed significantly higher coverage of known species. This difference highlights the importance of wildlife microbiota as a novel microbial resource and provides a new foundation for exploring host-specific microbial functions.

Primary BAs are initially synthesized in the liver and secreted into the intestine, where they are subsequently converted into secondary BAs through microbial processes such as bile salt hydrolysis and 7α-dehydroxylation (Hu, et al., 2022; McGavigan, et al., 2017; Peng, et al., 2024). In this experiment, we found that 73 % of the 1034 MAGs carried genes related to BA metabolism, covering key metabolic steps including bile salt hydrolysis (BSH), hydroxylation (such as 7α-HSDH), and 7α-dehydroxylation (such as baiCD). These findings reveal the broad functional potential of wild bird gut microbiota in BA metabolic pathways, suggesting that they may play an important role in host metabolic regulation(Li, et al., 2020). Specifically, microbial BA transformations have been demonstrated to play a pivotal regulatory role in host lipid absorption, glucose homeostasis, and energy metabolism by modulating the composition and size of the BA pool (Ridlon, et al., 2006; Wahlström, et al., 2016). Approximately 50 % of the MAGs encode BSH, indicating its high prevalence and potential functional importance in BA metabolism by the gut microbiota of wild birds. It's worth noting that BSH functions in wild birds are carried out by microbial taxa that are significantly different from those in humans and chickens. In wild birds, *Helicobacter_G* and *Ligilactobacillus* are the main bacterial genera responsible for bile salt hydrolysis, whereas *Collinsella* and *Alistipes* predominate in humans and chickens, respectively. These differences in microbial lineages suggest that the core function of bile salt hydrolysis may exhibit distinct host specificity, a pattern consistent with functional redundancy where evolutionarily diverse taxa perform the same key function (Foley, et al., 2021). This phenomenon of “functional redundancy” implies that, although carried out by different bacterial genera, the production of deconjugated bile acids is crucial for controlling intestinal pathogens, regulating cholesterol excretion, and initiating subsequent secondary bile acid formation (Guzior and Quinn, 2021). The critical nature of these functions underscores their potential as targets for nutraceutical and pharmaceutical interventions (Joyce and Gahan, 2016). Conversely, the shared functional potential between wild birds and chickens suggests convergent evolution of core metabolic roles, offering insights for manipulating microbiomes in poultry production. Furthermore, 20 % of the MAGs encode 7α-hydroxysteroid dehydrogenase (7α-HSDH), and 1.1 % harbor genes encoding BA-inducible enzymes such as baiCD. Notably, the relatively higher abundance of 7α-HSDH in the guts of wild birds compared to the other two groups indicates their potentially stronger metabolic capability in secondary BA biotransformation (Long, et al., 2017; You, et al., 2020). This finding may have important physiological implications. For example, microbial 7α-HSDH activity is closely associated with the bidirectional conversion of bile acids, thereby influencing the compositional balance of intestinal BAs (Ridlon, Kang and Hylemon, 2006). Moreover, secondary BAs, such as deoxycholic acid (DCA) generated by the bai gene cluster, function not only as potent antimicrobial agents but also as signaling molecules that can remotely modulate host hepatic metabolism and immune responses by activating nuclear receptors such as the farnesoid X receptor (FXR) (Campbell, et al., 2020; Sinha, et al., 2020). Therefore, we speculate that the strong BA-transforming potential exhibited by the gut microbiota of wild birds may be associated with their ecologically adaptive strategies, such as flight or migration. which are characterized by high energy demands. This perspective provides support for a novel dimension of host-microbe co-evolution in wild birds.

Previous studies have demonstrated that the residency status of animals can influence their gut microbiota composition and metabolic potential, particularly in functional pathways related to energy utilization and host adaptation (Risely, et al., 2018). This study identified significant differences in BA metabolic capabilities between two ecological types of wild birds: migratory birds (MB) and resident birds (RB). Through diversity analysis, we determined that the MB group exhibited higher diversity and composition of functional groups compared to the RB group. Due to their seasonal migratory activities, MB birds typically encounter more diverse and dynamic ecological environments (Gregory, et al., 2023). Their gut microbiota may enhance their ability to cope with external environmental changes through functional diversity. The two groups showed differences in BA biotransformation genes at both the phylum and genus levels, which may be related to differences in their host-associated microbiota (Grond, et al., 2019). These findings not only support the association between ecological behavior and metabolic potential from a microbial functional perspective but also provide new evidence for understanding the ecological evolution of bird gut microbiota. Apart from the influence of residency status, environmental factors significantly impact the composition of gut microbiota (Davenport, et al., 2014; Suzuki, 2017). This study found that climatic factors such as annual precipitation, temperature seasonality, and precipitation seasonality significantly affected the structural composition of BA biotransformation functional genes. In particular, precipitation seasonality played a crucial role in explaining variations within the RB group. These climatic variables not only influence host distribution and behavioral patterns, but may also indirectly shape the composition and structure of gut microbial communities by altering dietary sources and the intestinal physical environment (e.g., pH and permeability) (Kers, et al., 2018; Wongnak et al., 2022). Furthermore, species and diet showed significant effects. PERMANOVA analysis revealed that host identity explained over 34 % of the genomic functional variation, suggesting significant differences in microbial functional structures at the host level.

We acknowledge that certain limitations inherent in our meta-analytic approach should be considered. The limited availability of individual-level metadata such as age, sex, and reproductive status (Hird, 2017) precludes finer-scale analysis, although we have statistically accounted for host species as the major confounding factor. More importantly, the functional predictions generated from our metagenomic analysis, while robust, await direct experimental and metabolomic validation.

Nevertheless, these limitations precisely chart the course for future research. Building upon the genomic insights presented here, future studies should integrate metabolomic profiling to directly quantify bile acid profiles in wild birds. As emphasized in a comprehensive review of microbial bile acid transformations, combining metagenomics with metabolomics is crucial to bridge the gap between genetic potential and mechanistic understanding in host-microbe interactions (Guzior and Quinn, 2021). Integrating such multi-omics data with expanded sampling and hypothesis-driven experiments—including microbial cultivation, functional assays, and gnotobiotic models—will be essential to definitively verify the proposed mechanisms of BA transformation and advance our understanding of avian physiology.

## Conclusion

This study systematically analyzed the genomic functional characteristics of the gut microbiota in wild birds involved in BA metabolism, unveiling its uniqueness in terms of functional composition, taxonomic origin, and host specificity. From 10,455 MAGs, we screened and obtained 1,034 high-quality genomes, discovering that over 70 % of them carried key metabolic enzymes involved in BA transformation. Specifically, genes such as BSH and 7α-HSDH were widely distributed across multiple bacterial phyla and exhibited distinct host-specific genealogies in birds compared to humans and chickens. Further comparative analyses indicated significant differences between MB and RB populations in microbial community structure, BA metabolic potential, and functional group composition. Additionally, RDA and PERMANOVA analyses revealed that host species was the primary factor driving variations in BA metabolic gene composition, while environmental factors such as precipitation seasonality and annual precipitation also significantly influenced their distribution patterns. In summary, this study untangled the host-specific functional characteristics and ecological regulatory mechanisms of the gut microbiome in wild birds involved in BA metabolism, providing new insights into the functional contributions of wild bird gut microbiota to BA biotransformation and offering a new perspective on understanding host-microbe interactions in BA metabolism.

## CRediT authorship contribution statement

**Meng-Ting Yang:** Writing – original draft, Visualization, Software, Formal analysis. **Lin-Hong Xie:** Writing – review & editing, Funding acquisition, Conceptualization. **Ling Wang:** Writing – review & editing, Supervision, Resources. **Ying-Qian Gao:** Writing – review & editing, Resources. **Rui Liu:** Writing – review & editing, Visualization, Supervision. **He Ma:** Writing – review & editing, Supervision. **Cong-Cong Lei:** Writing – review & editing, Resources. **Jing Jiang:** Writing – review & editing, Validation, Supervision, Software, Conceptualization. **Jin-Wen Su:** Writing – review & editing, Formal analysis. **Xiao-Xuan Zhang:** Writing – review & editing, Visualization, Project administration. **Hong-Bo Ni:** Writing – review & editing, Visualization, Resources. **Fu-Long Nan:** Validation, Supervision, Software, Funding acquisition, Conceptualization.

## Disclosures

The authors declare that they have no known competing financial interests or personal relationships that could have influenced the work reported in this paper.

## Appendix Supplementary materials

Image, image 1

Image, application 2

## Footnotes

Supplementary material associated with this article can be found, in the online version, at doi:10.1016/j.psj.2025.105956.
