# Supplementary figures and images for: Metagenomic analysis of bile acid biotransformation by gut microbiota in wild birds
Source: Poult Sci. 2025 Oct 10;104(12):105956. doi: 10.1016/j.psj.2025.105956 (PMC12554960; doi:10.1016/j.psj.2025.105956)

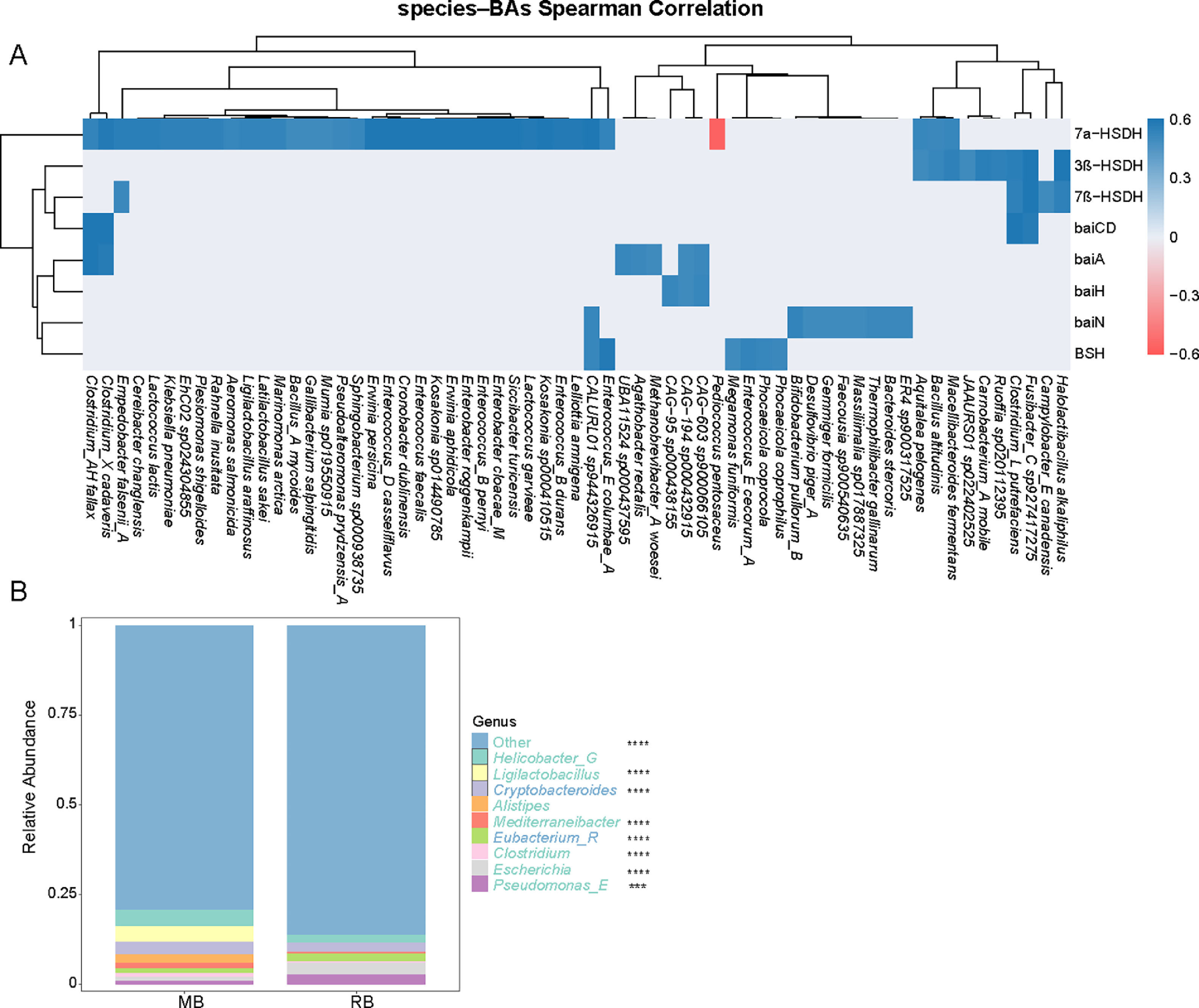

Supplement: Supplementary file 1 [file mmc1.jpg]
